# Supplementary material for: Long-term risk of death and recurrent cardiovascular events following acute coronary syndromes
Source: PLoS One. 2021 Jul 1;16(7):e0254008. doi: 10.1371/journal.pone.0254008 (PMC8248628; doi:10.1371/journal.pone.0254008)
Supplement: S1 Table — MI—myocardial infarction, NSTEMI—non-ST segment elevation myocardial infarction; STEMI—ST segment elevation myocardial infarction; MI—myocardial infarction; HF—heart failure; PVD- peripheral vascular disease; CVD—cerebrovascular disease; DAD—discharge abstract database; dx—diagnosis. (DOCX) [file pone.0254008.s001.docx]

**S1 Table. Variable definitions**

| **Condition** | **ICD 10 code** | **Dataset** | **Usage** |
| --- | --- | --- | --- |
| MI | I21, I22 | DAD, any dx | Outcome |
| Stroke | I63 | DAD, any dx | Outcome |
| Unstable angina | I200 | DAD, main dx | Inclusion |
| NSTEMI | I214 | DAD, main dx | Inclusion |
| STEMI | I210, I212, I212, I213 | DAD, main dx | Inclusion |
| Unspecified myocardial infarction | I219 | DAD, main dx | Inclusion |
| HF | I09.9, I11.0, I13.0, I13.2, I25.5, I42.0, I42.5, I42.6, I42.7, I42.8, I42.9, I43, I50, P29.0 | DAD, any dx | Covariate |
| Hypertension | I10, I11, I12, I13, I15 | DAD, any dx | Covariate |
| Diabetes | E10-E14 | DAD, any dx | Covariate |
| PVD | I70, I71, I73.1, I73.8, I73.9, I77.1, I79.0, I79.2, K55.1, K55.8, K55.9, Z95.8, Z95.9 | DAD, any dx | Covariate |
| CVD | G45, G46, I60-I69, H34.0 | DAD, any dx | Covariate |
| Renal disease | I12.0, I13.1, N03.2, N03.3, N03.4, N03.5, N03.6, N03.7, N05.2, N05.3, N05.4, N05.5, N05.6, N05.7, N18, N19, N25.0, Z49.0, Z49.1, Z49.2, Z94.0, Z99.2 | DAD, any dx | Covariate |
