# Supplementary material for: Long-term risk of death and recurrent cardiovascular events following acute coronary syndromes
Source: PLoS One. 2021 Jul 1;16(7):e0254008. doi: 10.1371/journal.pone.0254008 (PMC8248628; doi:10.1371/journal.pone.0254008)
Supplement: S2 Table — ACS—acute coronary syndromes; UA—unstable angina; NSTEMI—non-ST segment elevation myocardial infarction; STEMI—ST segment elevation myocardial infarction; CABG—coronary artery bypass grafting; PCI—percutaneous coronary intervention; ACEi—angiotensin converting enzyme inhibitor; ARB: angiotensin receptor blocker; MRA—mineralocorticoid receptor antagonist/aldosterone receptor antagonist. (DOCX) [file pone.0254008.s002.docx]

**S2 Table. In-Hospital Management of ACS**

|  | UA | NSTEMI | STEMI | Total |
| --- | --- | --- | --- | --- |
| Total N | 5135 | 15358 | 10563 |  |
| Coronary angiography at index | 3879 (75.5) | 11744 (76.5) | 9717 (92.0) | 25340 (81.6) |
| PCI within 30 days from admission | 2094 (40.8) | 7322 (47.7) | 8511 (80.6) | 17927 (57.7) |
| CABG within 30 days from admission | 399 (7.8) | 1231 (8.0) | 263 (2.5) | 1893 (6.1) |
| Thrombolysis at index | N/A | N/A | 2727 (25.8) | N/A |
| Discharge Medications within 3 months after discharge* | | | | |
| Total N | 4863 | 13623 | 9362 | 27848 |
| P2Y12 inhibitor | 2822 (60.4) | 9760 (75.3) | 7986 (89.9) | 20568 (77.5) |
| Anticoagulant | 372 (8.0) | 1303 (10.0) | 1637 (18.4) | 3312 (12.5) |
| Beta-blocker | 3574 (76.5) | 11127 (85.8) | 8090 (91.0) | 22791 (85.9) |
| ACEi/ARB | 3573 (76.5) | 10680 (82.4) | 8046 (90.5) | 22299 (84.1) |
| Statin | 3882 (83.1) | 11471 (88.5) | 8390 (94.4) | 23743 (89.5) |
| MRA | 158 (3.4) | 665 (5.1) | 682 (7.7) | 1505 (5.7) |
| Spironolactone | 158 (3.4) | 655 (5.1) | 649 (7.3) | 1462 (5.5) |
| Eplerenone | 0 (0.0) | 11 (0.1) | 36 (0.4) | 47 (0.2) |

* In patients who were discharged alive and survived at least 3 months after discharge. All comparisons were significant at p<0.01.
