# Supplementary figures and images for: Long-term risk of death and recurrent cardiovascular events following acute coronary syndromes
Source: PLoS One. 2021 Jul 1;16(7):e0254008. doi: 10.1371/journal.pone.0254008 (PMC8248628; doi:10.1371/journal.pone.0254008)

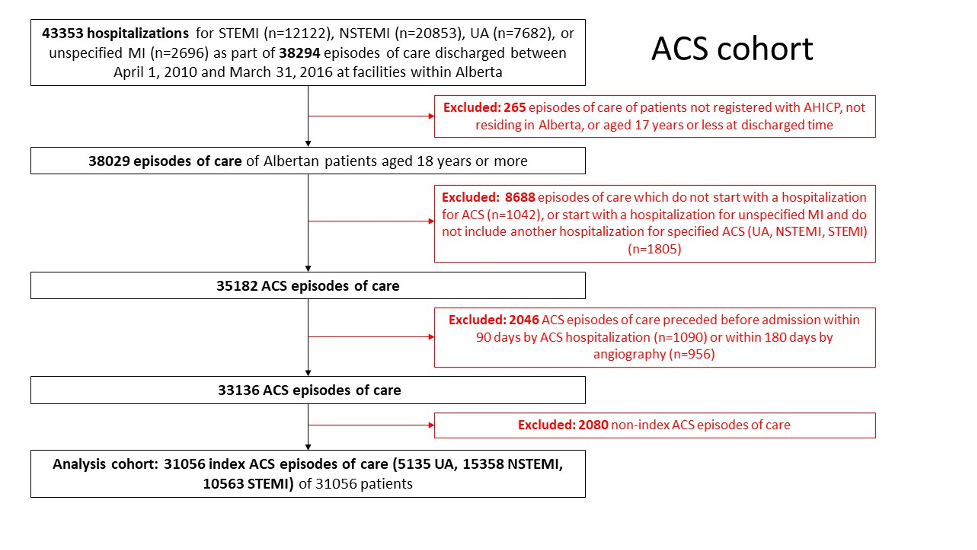

Supplement: S1 Fig — (TIF) [file pone.0254008.s003.tif]

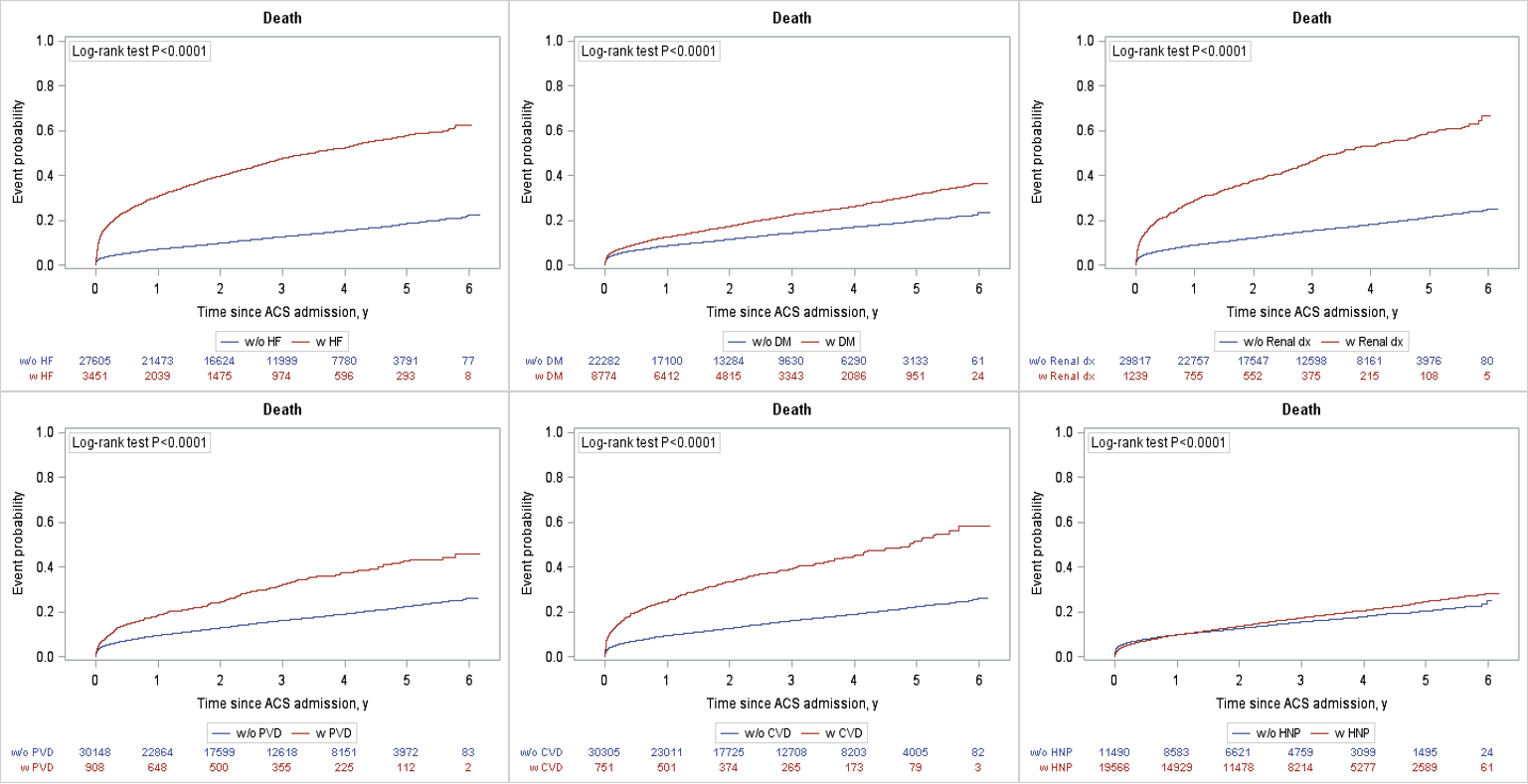

Supplement: S2 Fig — (TIF) [file pone.0254008.s004.tif]
